# Analysis of composition and microstructure of diatom frustules in mud on the coast of Boryeong- city, South Korea

**DOI:** 10.1186/s42649-022-00082-1

**Published:** 2022-12-15

**Authors:** Mi Kyung Bok, Chung Hwa Chin, Hee Jung Choi, Ju Hyun Ham, Byung Soo Chang

**Affiliations:** 1 Pango Korea Co., Ltd. Boryeong, 33443 Chungnam, South Korea; 2 Department of Global Medical Beauty, Konyang University, 32992 Nonsan, Chungnam South Korea; 3 Department of Public Health, Graduate School of Integrative Medicine, CHA university, 13488 Seongnam, Gyeonggi-do South Korea; 4 Department of Health Consultation Welfare, Hanseo University, Chungnam 31962 Seosan, South Korea; 5 Department of Cosmetology, Hanseo University, 31962 Seosan, Chungnam South Korea

**Keywords:** Mud, Clay mineral, Diatom, Frustule, EDS, SEM

## Abstract

The microstructure of diatom frustules found in mud sediments along the coast of Boryeong- city, South Korea, was observed using a scanning electron microscopy (SEM), and the constituent elements of diatoms were analyzed using energy**-**dispersive X-ray spectroscopy (EDS). Diatom frustules and clay minerals were present in the SEM images of the mud powder. High-magnification SEM images revealed that the surface of the frustules contained identically shaped circular pores, measuring 1 μm in diameter, arranged at regular intervals. This study revealed that the diatom shell fragments in the mud powder ranged in size from 3 to 30 μm, with an average thickness of approximately 2.5 μm. The elements Si, Al, Fe, K, Na, Mg, and Ti were detected while analyzing the frustule constituents, with Si being the primary component with the highest content.

## Introduction

In South Korea, mud is a naturally occurring substance found along the coasts of Boryeong, Muan, Taean, and Pohang- cities. By developing products using mud and holding skin beauty-related events regularly, the local government remarkably contributes to the health care and healing of tourists.

Mud is a viscous, naturally occurring, fine-grained suspension commonly observed on tidal flats. It is made up of clay minerals, water, sand, silt, and small amounts of organic matter of different origins and compositions (Berlamont et al. 1993; Schindler et al. 2015; Shakeel et al. 2019).

Recently, clay minerals have been the subject of numerous studies in various fields, such as geology, cosmetology, materials science, pharmacy, medicine, food science, and biotechnology. They have been used as traditional folk remedies over the last 2,000 years because of their health benefits. With the development of modern science and technology, they are being utilized in traditional healing therapies and other natural skin care products (De Vos 2010; Kim et al. 2016; Kamitsou et al. 2018).

The mud obtained from nature is a semi-solid mixture of various minerals, organic substances, including diatoms, and water. The three forms of mud application used to treat rheumatic and skin diseases are mud packs, mud wraps, and mud baths (Gomes et al. 2013).

Mud may cause swelling over large, specific surface areas because of its colloidal particle size, high cation exchange capacity, and semi-solid properties (Moraes et al. 2017; Khiari et al. 2019).

The rheological properties of mud are a combination of yield stress, thixotropy, and viscoelasticity, which are typically caused by the bonding of hard particles, such as clay minerals, with soft polymers, such as organic materials. Natural fine particle suspensions in mud typically exhibit complex rheological properties because of the presence of clay minerals and organic matter. Its rheological properties may vary from region to region due to variations in the organic content of its composition (Shakeel et al. 2021).

Van Kessel and Blom (1998) compared the rheological properties of pure clay minerals, such as kaolinite, and mud samples and discovered that the mud samples produced higher yield stress values. This finding was attributed to the presence of organic substances in the mud sample.

The rheological properties of mud samples depend on their density, and even small amounts of organic matter can significantly affect the mud’s rheological behavior (Kranenburg 1994; Van Kessel and Blom 1998; Shakeel et al. 2019). Diatoms are unicellular, eukaryotic, photosynthetic algae found in aquatic environments. They have significant ecological importance on this planet and exist as frustules in mud deposits. They are a type of microalgae with sizes ranging from 10 to 200 μm. They grow suspended or attached in freshwater or seawater where there is light, nutrients, and moisture (Leonardo et al. 2017).

Bok et al. (2021) reported the fine structure of clay minerals present in the mud powder produced in Boryeong- city, South Korea, but no research on diatoms contained in these clays has been published. This study contributes to the development of mud-based cosmetics by validating the homogeneity and composition of mud particles used in various health care and beauty products.

In this study, the microstructural characteristics of diatoms present in the mud powder taken from the coast of Boryeong- city, South Korea, were observed using scanning electron microscopy (SEM), and their constituent elements were analyzed using energy**-**dispersive X-ray spectroscopy (EDS).

## Materials and methods

### Sample processing

Dry, sterilized mud powder was purchased from Boreyung MUD+ (Cosmax Co., Korea). The raw mud was ball-milled to produce commercial mud, which was harvested from tidal flats along the coast of Boryeong-city, South Korea.

To observe the microstructural characteristics and analyze the constituent elements of the mud powder, 1 g of sterilized mud powder was placed in a 50-ml Falcon tube, and 30 ml of absolute alcohol (Merck Co., Germany) was added. Then, the sample was washed three times with alcohol for 1 h each to allow for precipitation.

### Scanning electron microscopy observations

A sample of approximately 0.1 g of mud powder was deposited on a stub treated with carbon and copper tape and then dried in a vacuum dryer (60℃, DPF-31, Yamato, Japan) for 24 h. Then, the platinum was plated to a thickness of 20 nm using an ion deposition machine (IB-5 ion coater, Eiko, Japan), and the sample was observed at 15 kV with SEM (S-4700, Hitachi, Japan).

### Energy-dispersive X-ray spectroscopy analysis

To analyze the constituent elements of diatom frustules contained in the mud powder, the sample was treated similarly to the SEM experiment and then placed on a support with carbon tape and coated with platinum to a thickness of 20 nm using an ion coater (IB-5, Eiko, Japan). The coated sample was analyzed using EDS at an acceleration voltage of 15 kV (INCA, Oxford Ins., Great Britain).

## Results

**Fig. 1:**
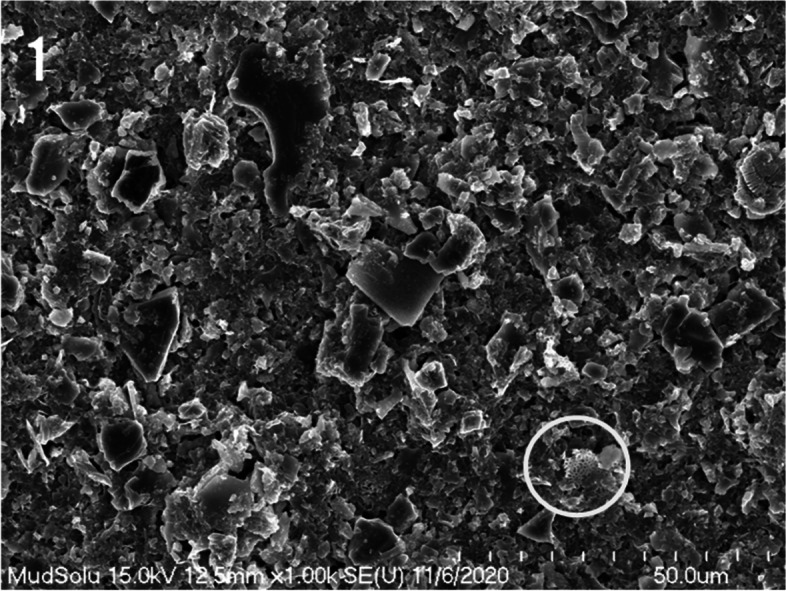
A scanning electron micrograph of mud precipitation depicting microorganism remnants (circle)

In the SEM observation of the mud powder, substances presumed to be remnants of marine microorganisms, excluding clay minerals, were observed. These residues form fine circular holes arranged at regular intervals (Fig. 1). The mud powder was mostly composed of fine particles and crystalline minerals. The surface of the crystalline clay minerals was noted to be smooth.

In Fig. 1, the remnants of marine microorganism were split into fine fragments and surrounded by mud particles of varying sizes and shapes.

Relatively large mud particles appeared in the form of crystals. The plate-like or cubic crystalline clay minerals have a remarkably smooth and flat surface. Clay minerals of various sizes and shapes were scattered throughout the mud powder (Fig. 1).

**Fig. 2:**
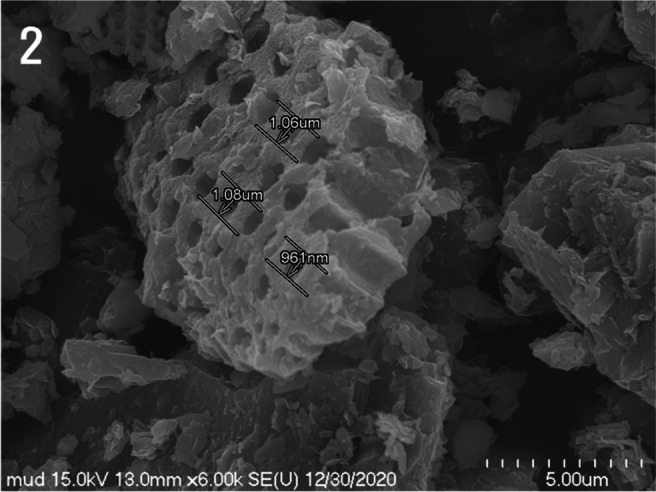
Magnified scanning electron micrograph of Fig. 1 depicting the diatom frustule

**Fig. 3:**
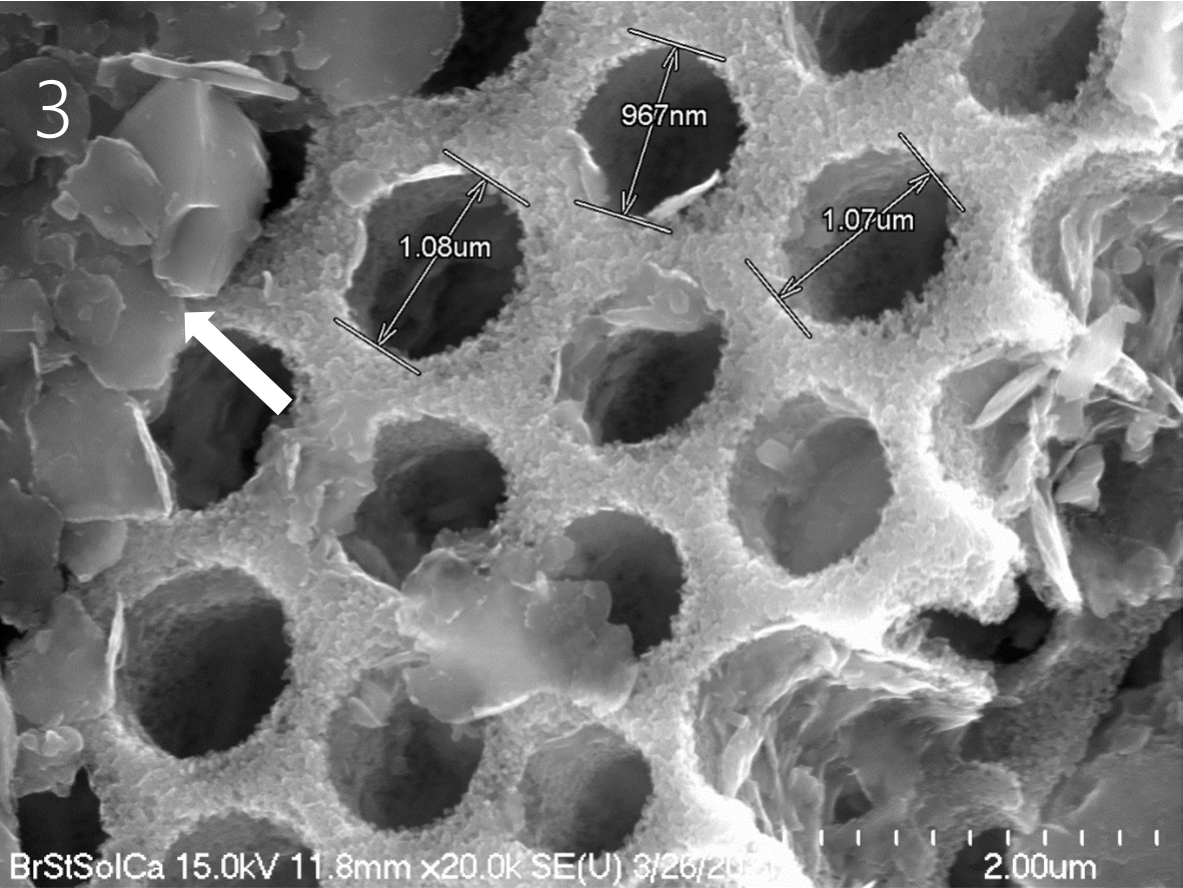
High-magnification scanning electron micrograph of uniformly arranged circular pores of the frustule in diatom remnants. The areolae are arranged in regular patterns across the frustule surface. Arrow: Plate-shaped clay minerals

The fragmented remains of marine microorganisms were identified as the frustules of diatoms from high-magnification SEM images. The frustule fragments had a lateral thickness of approximately 2.5 μm and displayed uniform circular holes with sizes ranging from approximately 961 nm to 1.08 μm (Figs. 2 and 3). A plate-shaped clay mineral with a smooth surface was attached to the surface of the shell (Fig. 3).

**Fig. 4:**
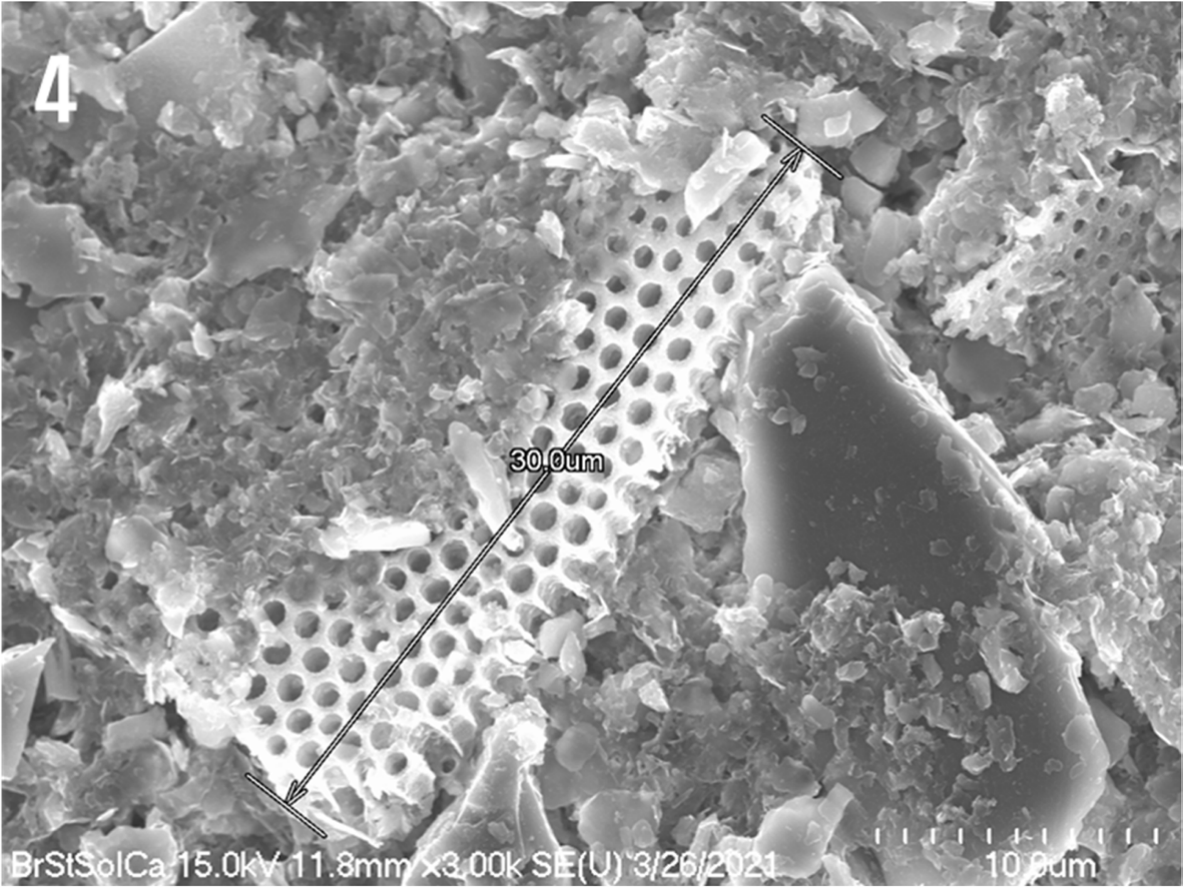
Scanning electron micrograph of the complex hierarchical and well-maintained inherent porous structure of a diatom frustule

In Fig. 4, depicts fragments of diatom frustules with evenly spaced holes up to 30 μm in length. These diatom frustules were mixed with clay minerals in the mud powder. It included both large and small mineral particles in the vicinity of the diatom frustules, as well as crystalline minerals with a diameter of approximately 25 μm.

**Fig. 5:**
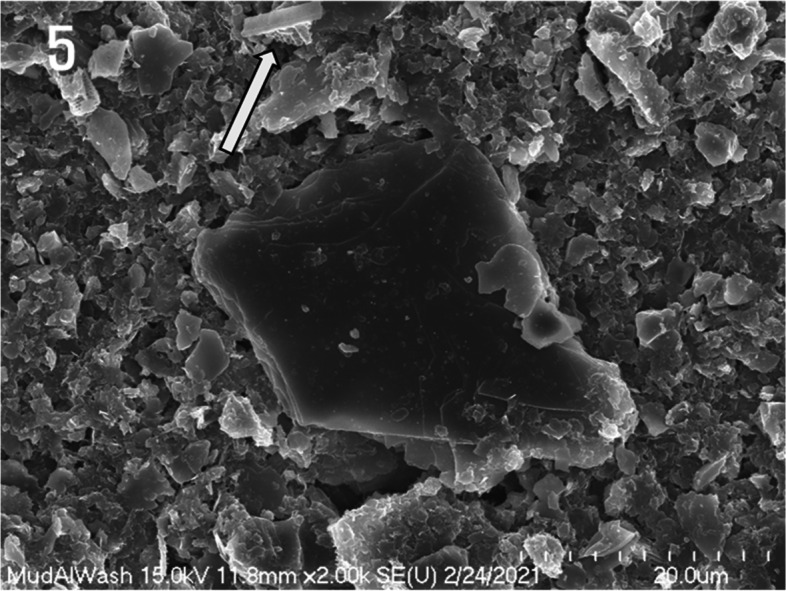
Scanning electron micrograph of mud powder depicting a diatom frustule fragment (arrow) and clay mineral

In Fig. 5, the diatom shell edge is fractured, and the edge interface is extended in a straight line. Clay crystals with a diameter of approximately 38 μm were also observed, along with fine clay minerals.

**Fig. 6:**
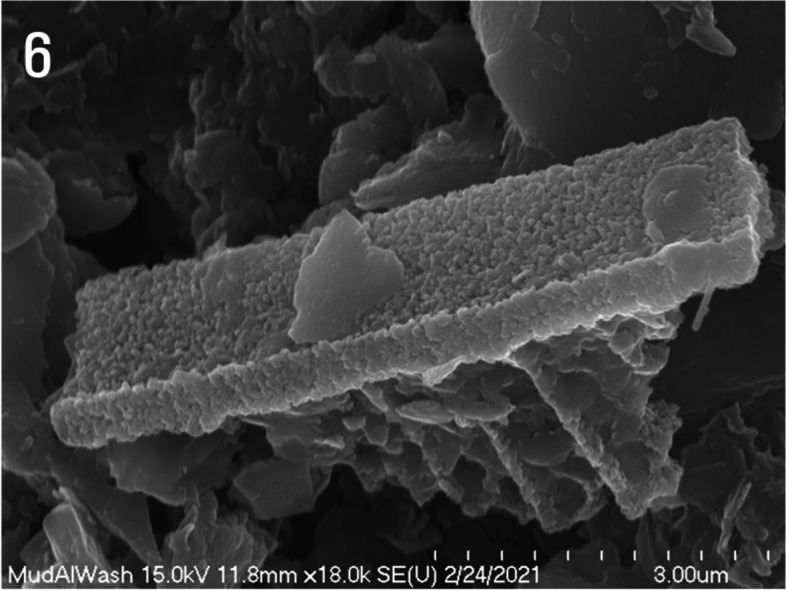
High-magnification scanning electron micrograph of a silica frustule with a thickness of to 2.5 μm

According to high-magnification SEM images, the diatom frustule’s outer shell boundary surface was not smooth; continuous formation of minute irregularities occurred, and the thickness was approximately 2.5 μm. Additionally, the inner portion of the outer circumferential surface revealed traces of circular pores (Fig. 6).

**Fig. 7:**
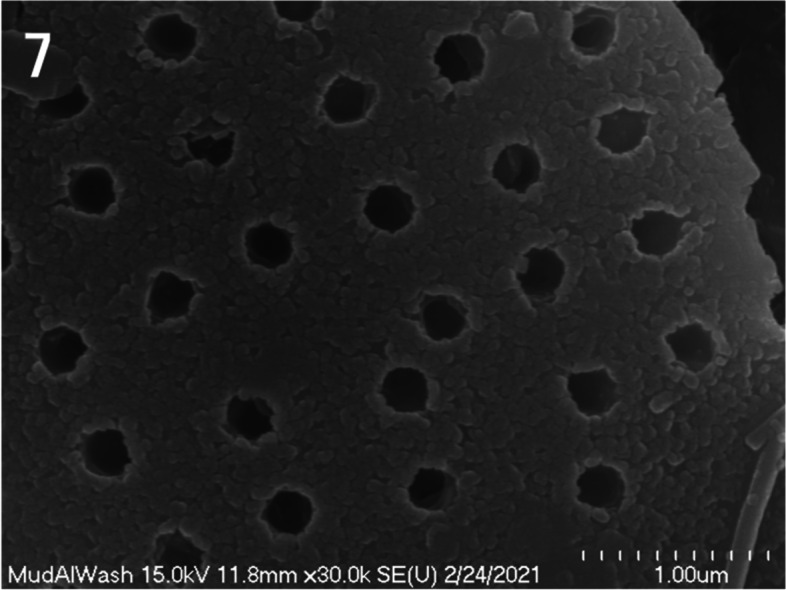
Scanning electron micrograph of the diatom frustule. Note that the edge of the diatom frustule is circular and the pores are uniformly arranged on the surface

As depicted in Fig. 7, the edges of diatom shells are curved, and the pores on the frustule surface are arranged at regular intervals.

**Fig. 8:**
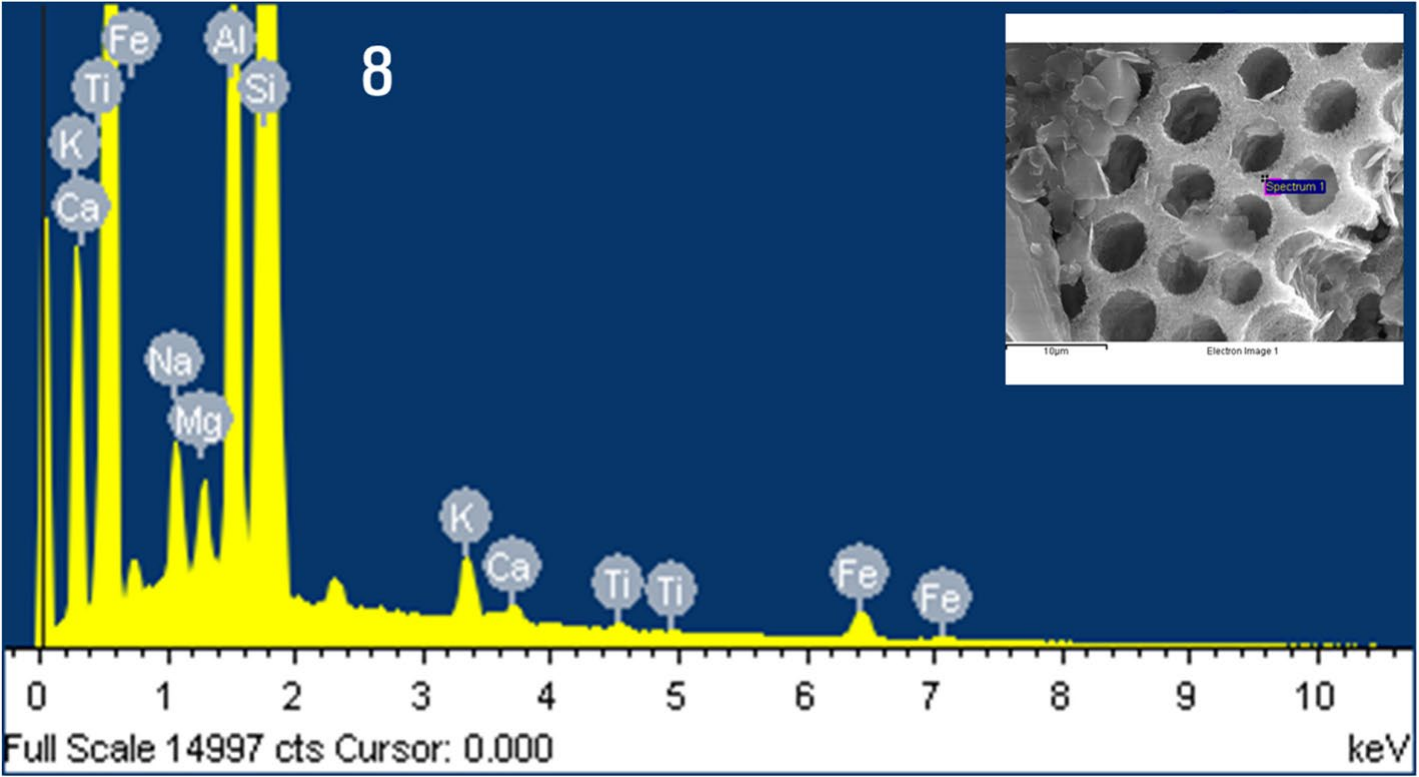
Energy-dispersive X-ray spectroscopy (EDS) spectrum of the diatom frustule. EDS reveals that Si, Al, Fe, K, Na, Mg, and Ti elements are present on the diatom frustule. Inset: Scanning electron micrograph of the mud powder displaying the detection area of the EDS spot range

The constituent elements of the diatom frustule present in the mud powder were analyzed using EDS. After verifying the analysis site using an SEM with high magnification and precise focusing, the detection site was identified and analyzed using an EDS (Fig. 8). As a result of analyzing the components of the diatom frustule, an analysis pattern with Si, Al, Fe, K, Na, Mg, and Ti element peaks was confirmed (Fig. 8). The content of each of these elements was detected in the following order: Si element 72.99% > Al element 10.67% > Fe element 5.83% > Mg element 5.31% > K element 2.95% > Na element 1.78% > Ti element 0.45% (Table 1). The majority of the diatom shell’s constituent elements were Si elements.

**Table 1:** Energy-dispersive X-ray spectroscopy analysis of the elemental composition of the diatom frustule of mud powder

Element	Weight%	Atomic%
**Na K**	1.38	1.78
**Mg K**	4.35	5.31
**Al K**	9.69	10.67
**Si K**	69.00	72.99
**K K**	3.89	2.95
**Ti K**	0.73	0.45
**Fe K**	10.96	5.83
**Total**	**100.00**	

## Discussion

In this study, fragments presumed to be marine microbial remnants, excluding clay minerals, were discovered in the mud powder. These microflakes were observed in various sizes depending on the degree of fragmentation. The fragments possessed a plate-like structure with a thickness of 2.5 μm and a length of up to 30 μm, with many circular pores formed at regular intervals. Circular pores with a diameter of approximately 1 μm were arranged in a regular pattern on the surface.

Baschini et al. (2010) reported that natural sulfur-rich mud has amorphous siliceous microbial, most of which are diatom frustules. The shell and cell wall of diatoms have a colorful shape and design and are made of amorphous hydrated silica (SiO2–(H2O)n) produced by cell biomineralization (Habchi et al. 2006). Diatoms belonging to the genus *Coscinodiscus* are single-celled algae that form a silica shell or frustule with pores of 1 μm or less arranged at regular intervals (Jeffryes et al. 2008; Zaman et al. 2020).

Diatoms are unicellular photosynthetic plants that live in both freshwater and seawater, and their shells are composed of amorphous biosilica components with pores that are systematically and uniformly arranged (Xing et al. 2017). As reported by Baschini et al. (2010), the remnants of marine microorganisms were identified as typical diatom shells in this study. The pores were uniformly and systematically arranged across the holes on the frustule surface.

Additionally, high-magnification SEM images revealed that the shell’s surface was composed of amorphous biosilica with some irregularities. The formation of surface irregularities is caused by the accumulation of silica in the form of fine granules as the mineralization progresses.

Diatom frustules with such microstructural characteristics and silica chemical composition have been used in various industrial applications. Additionally, the porous cytoskeleton’s, excellent physical filtration and transportation capabilities have been utilized in various studies, such as light harvesting through needle-like pores, photon and molecule separation, and drug delivery (Hale and Mitchell 2001; Jeffryes et al. 2008; Gnanamoorthy et al. 2014; Kieu et al. 2014; Dong et al. 2016).

The organic compounds, ions, and inorganic soluble complexes in the mud powder serve as physicochemical factors that determine the efficacy of spa therapies (Fioravanti et al. 2011). Organic compounds accumulate in the mud following the formation of cyanobacteria, a type of blue-green algae. The organic molecules secreted by these microorganisms include glycolipids and sulfoglycolipids. Organic have anti-inflammatory properties and play a crucial role in mud aggregation (Sukenik et al. 1999; Tolomio et al. 1999; Carretero et al. 2010; Trabelsi et al. 2016). In the SEM observation of the mud powder employed in this study, cyanobacteria and photosynthetic prokaryotes were not observed; however, diatoms were detected as fine fragments with pulverized shells.

It is impossible to examine the microstructure of cyanobacteria and bacteria in the mud mixture samples because the mud collected from the shore has a high degree of purity and is heat sterilized, milled, and wet separated for convenience (Viseras et al. 2007). It is considered that their morphological characteristics cannot be validated due to the decomposition or separation of the organic components.

Analyzing the constituent elements in the diatom shell, this research detected traces of Si, Al, Fe, K, Na, Mg, and Ti but not Ca and S, which are common in clay minerals.

Si was the frustule’s primary constituent element, with a content of 72.99%. Al was detected with a content of 10.67%, along with trace levels of Fe, K, Na, Mg, and Ti. The Si content in the diatom shell was higher than that in the entire mud powder. This result indicates that silica is the main component of the diatom frustule. It is considered that the detected elements, excluding Si, are caused by the influence of clay mineral particles present in trace amounts in the analysis sample.

There are three different types of silica sources for marine sediments. First, land-influent silica, which is primarily produced by land-based materials entering rivers; second, bio-derived silica, primarily obtained from biological structures or skeletal remnants of microorganisms and secretions of siliceous organisms; and third, hydrothermal silica, typically associated with hydrothermal fluids (Xu et al. 2021).

## Conclusion

Diatom frustules were observed in mud sediments off the coast of Boryeong- city, South Korea. They have a plate-like structure, with circular pores of approximately 1 μm in diameter distributed at regular intervals on the frustule surface. The size of the frustule fragments observed in this study ranged from approximately 4–30 μm, with a thickness 2.5 μm. Si, Al, Fe, K, Na, Mg, and Ti were detected were detected in the diatom shell, and Si was confirmed to be the main component, accounting for approximately 73% of the shell’s composition.

## Data Availability

Data and materials are available on request.

## Abbreviations

EDS: Energy-dispersive X-ray spectroscopy
SEM: Scanning electron microscope
